# Association between the triglyceride-glucose index and silent myocardial infarction in the general population

**DOI:** 10.1038/s41598-025-92779-y

**Published:** 2025-03-12

**Authors:** Min Dai, Dongze Li, Jing Yu, Yi Liu, Qin Zhang, Wei Zhang, Yan Zhong, Zhi Wan, Menglin Tang, Yongli Gao, Li Rao

**Affiliations:** 1 Department of Emergency Medicine, West China Hospital, Sichuan University/West China School of Nursing, Sichuan University, 37 Guoxue Lane, Chengdu, 610041 Sichuan China; 2 Department of Cardiac Surgery, West China Hospital, Sichuan University/West China School of Nursing, Sichuan University, 37 Guoxue Lane, Chengdu, 610041 Sichuan China; 3 Department of Geriatric Nursing, Sichuan Provincial People’s Hospital, University of Electronic Science and Technology of China, No. 32 West Second Section, First Ring Road, Chengdu, 610072 Sichuan China

**Keywords:** Silent myocardial infarction, Cardiovascular risk factors, Triglyceride glucose index, Health care, Medical research

## Abstract

This study aimed to explore the relationship between the triglyceride-glucose (TyG) index and silent myocardial infarction (SMI) in the general population, with a focus on elucidating potential links and contributing to the understanding of risk factors for undetected cardiac events. This prospective cohort study was carried out within a community-based population, using data from the Atherosclerosis Risk in Communities study in the United States. The dataset included information on demographics, cardiovascular risk factors, blood lipids, liver and kidney function, and other variables. Participants were categorized into four quartiles based on their TyG index scores. Cox regression analysis was used to examine the relationship between the different ranges of TyG indices and SMI. In total, 14,211 community residents were enrolled and followed up for 36 years. Among them, 7,316 (51.48%) developed SMI. TyG index measurements were divided into quartiles: Q1 (≤ 8.26), Q2 (8.26–8.62), Q3 (8.62–9.02), and Q4 (≥ 9.02). Restricted cubic spline curves indicated that higher TyG indices correlated with a greater risk of SMI. Results of the Kaplan–Meier analysis suggested that participants with a higher TyG index had a lower cumulative survival rate for SMI (*P* < 0.001). Through multivariate Cox regression analysis, the TyG index was identified as an independent predictor of SMI risk (*P* < 0.001). Further stratified analyses reinforced the link between the TyG index and the risk of SMI, demonstrating its consistent influence across diverse population subsets. Mediation analysis revealed significant effects of hypertension, diabetes, body mass index and sex on SMI risk. The overall effect sizes ranged from 1.77 to 1.95, with direct effects accounting for 52.7–99.6% and mediation effects ranging from 0.4 to 47.3%. In the general population, the TyG index as an independent predictor of SMI risk, emphasizing its importance in cardiovascular disease assessment.

## Introduction

Silent myocardial infarction (SMI), which often presents without obvious symptoms like chest pain or shortness of breath, is commonly overlooked or misdiagnosed, making accurate detection challenging in the general population^1–4^. SMI is responsible for about half of all myocardial infarctions, with prevalence rates ranging from 22 to 60%^1,5,6^. Because SMI has insidious characteristics, it is easy to delay treatment. Studies have revealed that mortality in patients with SMI is three times higher than that in patients with acute MI^7–9^, hinting at a potentially robust association between SMI and the elevated risk of cardiovascular incidents as well as mortality. Given the high incidence and serious outcomes of SMI, early screening and identification of high-risk populations are essential for its prevention and management.

Prior investigations have revealed that individuals with diabetes exhibit a notably elevated predisposition to SMI, with a prevalence rate as high as 44.3%^10^. However, a history of diabetes only reflects the clinical background and does not allow for dynamic monitoring and evaluation. The main clinical features of diabetes include impaired glucose and lipid metabolism, with insulin resistance (IR) being a key underlying factor^11^. Therefore, we hypothesize that IR may be a significant factor contributing to the increased risk of SMI in diabetic patients^12^ and could serve as an effective indicator for the early identification of SMI. However, routine monitoring of IR levels is challenging due to the difficulty of rapid quantification. Hence, the identification of straightforward markers for the dynamic evaluation of IR holds paramount importance for the timely recognition of individuals at heightened risk for SMI, facilitating early intervention and improved patient outcomes.

The triglyceride-glucose (TyG) index serves as a straightforward marker for IR. A robust association has been noted between the TyG index and IR, with the TyG index exhibiting high diagnostic precision for IR. Prior studies have reported an area under the receiver operating characteristic curve of 0.858, further substantiating its accuracy in identifying with IR^13–17^. Investigations have additionally unveiled a tight connection between the TyG index and the vulnerability to both angina and acute myocardial infarction, emphasizing its potential as a predictive marker for adverse cardiovascular outcomes^18,19^. Notwithstanding the aforementioned, the investigation into the nexus between the TyG index and the risk of SMI in the general population remains scant. Hence, the present study endeavors to delve into the relationship between TyG levels and SMI risk through a prospective cohort analysis, with the ultimate objective of ascertaining the potential utility of the TyG index as a discerning marker for pinpointing individuals who are predisposed to a heightened SMI risk.

## Methods

### Study design and participants

This research utilized data from the Atherosclerosis Risk in Communities (ARIC) study; the database is managed by the Biological Samples and Data Storage Information Coordinating Center under the National Heart, Lung, and Blood Institute^20^. The ARIC is a multicenter prospective cohort analysis in a US community-based population with the primary aim of exploring risk factors associated with cardiovascular and cerebrovascular disease. The data were collected from 15,792 residents in four communities in the United States between 1987 and 2022. Follow-up was performed every 3 to 4 years through 2022, with 10 follow-ups over 36 years. The protocol of this study underwent rigorous scrutiny and was granted approval by the ethics review boards of the collaborating community-based research institutions and the Human Ethics Committee of West China Hospital, Sichuan University (approval number 2021 [1631]). All research activities were conducted in strict adherence to the ethical guidelines outlined in the Declaration of Helsinki, ensuring the protection of participants’ rights and well-being. All participants provided written informed consent.

The study was designed as a prospective cohort study, including a community population initially enrolled in the ARIC study. The specific inclusion criteria were: (1) participants aged between 45 and 64 years, and (2) long-term residents of the study community. Participants were followed over time to observe and assess the occurrence of SMI. Exclusion criteria: (1) Lack of key indicators such as blood sugar, triglycerides, etc.; (2) Total number of dropouts. Ultimately, the study included 14,211 participants who met all the eligibility criteria and were monitored prospectively for the occurrence of new SMI events.

### TyG index calculation

The TyG index was derived from baseline fasting triglyceride and plasma glucose levels. The calculation was performed using the formula: triglycerides (mg/dL) × fasting plasma glucose (mg/dL)/2, where 1 dL is equivalent to 0.1 L. TyG index measurements were divided into quartiles: Q1 (≤ 8.26), Q2 (8.26–8.62), Q3 (8.62–9.02), and Q4 (≥ 9.02). The division into quartiles was chosen to categorize the data into four equal groups, ensuring an even distribution of participants across the TyG index range.

### Data collection

In the ARIC study, various data, including demographic information, vital signs, smoking and alcohol consumption status, routine blood parameters, lipid-related markers, blood glucose levels, and renal function indicators, were collected at enrollment. Follow-up assessments were conducted every 3–4 years. Additional data gathered encompassed details pertaining to lifestyle patterns, hospital admissions, medical diagnoses and therapeutic interventions, as well as the occurrence of cardiovascular occurrences, such as coronary artery disease and congestive heart failure. Hyperlipidemia was characterized by a serum triglyceride level exceeding 1.7 mmol/L, total cholesterol levels higher than 5.72 mmol/L, or low high-density lipoprotein levels below 0.9 mmol/L.

### Research indicators

The main outcome of the study was the occurrence of SMI. The diagnosis of SMI is based on the detection of changes in baseline and follow-up electrocardiograms (ECGs), where the baseline ECG shows no evidence of myocardial infarction, but new Q waves, ST segment abnormalities, or other typical signs of myocardial infarction appear in subsequent follow-up ECGs. Additionally, participants must not have reported any symptoms associated with myocardial infarction, such as chest pain or shortness of breath, nor have any recorded diagnosis of myocardial infarction during the baseline and follow-up periods^21,22^. Electrocardiographic evidence of MI was determined based on the Minnesota Code (MC), which includes major Q/QS abnormalities (MC 1.1 or MC 1.2) or minor Q/QS abnormalities (MC 1.3) combined with major ST-T abnormalities (MC 4.1, MC 4.2, MC 5.1, or MC 5.2)^21,22^.

### Statistical analysis

In this study, Categorical variables have been depicted through frequencies (%) and were subjected to group-wise comparisons employing the chi-squared test. Meanwhile, continuous variables are represented as means with accompanying standard deviations (X ± S) and were compared utilizing either the t-test or ANOVA. For variables exhibiting non-normal distributions, comparisons were facilitated by employing the median along with the interquartile range or resorting to the Mann-Whitney U test for statistical analysis. Furthermore, to evaluate the potential nonlinear association between the TyG index and the risk of SMI, restricted cubic spline curves were implemented. Kaplan-Meier survival analysis was used to analyze differences in cumulative survival among populations with varying SMI levels across the TyG index population. Cox proportional hazards regression models were employed to assess the relationship between varying levels of the TyG index and the risk of SMI. To further dissect this association, subgroup analyses were performed, examining the interplay between the TyG index and SMI risk across various subgroups, encompassing sex, age cohorts, body mass index (BMI) categories, smoking and drinking status, as well as the presence of hyperlipidemia, diabetes, and hypertension. Mediation analysis was also performed to investigate the indirect effects of potential mediators on the relationship between the TyG index and SMI risk. The analysis aimed to quantify the extent to which variables such as sex, BMI, hypertension, diabetes, and hyperlipidemia mediated this association. The total, direct, and indirect effects of these factors were estimated using statistical models to assess their mediating influence.The SPSS version 26.0 and R version 4.3.0 was adopted.

## Results

### Participant baseline demographics and clinical profiles stratified by the TyG index levels

This study included 14,211 community residents, among whom 7316 (51.48%) cases of SMI were observed during the follow-up. The participants were divided into four distinct quartiles (Q1 to Q4) according to their TyG index values: Q1 (*n* = 3,553), Q2 (*n* = 3,554), Q3 (*n* = 3,551), and Q4 (*n* = 3,553). Individuals in the higher TyG index quartiles exhibited higher age, BMI, heart rate, and severity of hypertension and diabetes, as well as increased levels of glucose, triglycerides, hemoglobin, white blood cells, platelets, neutrophils, lymphocytes, total cholesterol, low-density lipoprotein, and uric acid. Conversely, the lower TyG index quartiles (Q1 and Q2) were characterized by a higher proportion of female participants and higher rates of alcohol consumption. Additionally, these groups showed elevated levels of high-density lipoprotein and creatinine. The differences across all groups were statistically significant (*P* < 0.05) (Table 1).

**Table 1:** Baseline characteristics of participants based on TyG index.

Baseline indicator	Q1 (*n* = 3553)	Q2 (*n* = 3554)	Q3 (*n* = 3551)	Q4 (*n* = 3553)	*p*-value
sex, Male	1285 (36.2)	1525 (42.9)	1736 (48.9)	1903 (53.6)	< 0.001
Age, years	52.0 [48.0,58.0]	54.0 [49.0, 59.0]	55.0 [50.0, 60.0]	55.0 [50.0, 60.0]	< 0.001
BMI, kg/m²	24.8 [22.4, 27.8]	26.1 [23.5, 29.4]	27.3 [24.5, 30.8]	28.9 [26.08, 32.4]	< 0.001
HR, bpm	64 [58.00, 71.00]	65 [59.00, 72.00]	66 [59.00, 73.00]	68 [62.00, 76.00]	< 0.001
SBP, mmHg	115 [104.00,127.00]	117 [107.00, 130.00]	120 [109.00, 132.00]	123 [113.00, 136.00]	< 0.001
DBP, mmHg	71 [65.00, 79.00]	73 [65.00, 80.00]	73 [67.00, 80.00]	74 [68.00, 81.00]	< 0.001
Drinking	2083 (58.6)	2029 (57.1)	1983 (55.8)	1943 (54.7)	< 0.001
Smoking	1859 (52.3)	2089 (58.8)	2169 (61.1)	2245 (63.2)	< 0.001
Hypertension	661 (18.6)	868 (24.4)	1053 (29.7)	1506 (42.4)	< 0.001
Diabetes	55 (1.5)	117 (3.3)	195 (5.5)	978 (27.5)	< 0.001
Hyperlipidemia	835 (23.5)	1267 (35.6)	2013 (56.7)	3283 (92.4)	< 0.001
Glu, mmol/l	5.19 [4.92, 5.50]	5.40 [5.11, 5.77]	5.61 [5.24, 6.00]	6.05 [5.55, 7.55]	< 0.001
Triglycerides	0.73 [0.62, 0.82]	1.06 [0.97, 1.17]	1.49 [1.33, 1.66]	2.25 [1.92, 2.78]	< 0.001
TC	5.04 [4.47,5.66]	5.39 [4.78, 6.03]	5.66 [5.02, 6.31]	5.90 [5.17, 6.65]	< 0.001
HDL	1.57 [1.29,1.89]	1.34 [1.12, 1.62]	1.19 [0.98, 1.42]	1.02 [0.85, 1.24]	< 0.001
LDL	3.08 [2.54, 3.67]	3.48 [2.92, 4.13]	3.74 [3.10, 4.39]	3.75 [3.10, 4.44]	< 0.001
WBC	5.20 [4.40, 6.40]	5.70 [4.70, 6.90]	5.90 [5.00, 7.20]	6.40 [5.40, 7.60]	< 0.001
Neutrophils	2.67 [1.57, 3.62]	2.80 [1.38, 3.89]	2.92 [1.12, 4.07]	3.08 [0.00, 4.28]	< 0.001
Lymphocytes	1.60 [1.15, 2.04]	1.66 [1.09, 2.15]	1.71 [0.97, 2.18]	1.77 [0.00, 2.27]	< 0.001
Platelets	249.00 [212.00, 289.00]	253.00 [216.00, 297.75]	252.00 [216.00, 299.00]	251.00 [212.00, 296.00]	0.001
Hemoglobin	13.40 [12.50, 14.30]	13.80 [12.90, 14.70]	14.10 [13.10, 15.00]	14.30 [13.40, 15.30]	< 0.001
Creatinine	106.23 [97.75,114.17]	102.94 [95.08, 111.08]	101.57 [93.60, 109.55]	101.16 [92.23, 109.18]	< 0.001
Uric acid	5.20 [4.40,6.20]	5.70 [4.80, 6.70]	6.20 [5.20, 7.10]	6.60 [5.60, 7.70]	< 0.001

Note: BMI: body mass index; SMI: silent myocardial infarction; HR: heart rate; SBP: systolic blood pressure; DBP: diastolic blood pressure; TC: total cholesterol; HDL: high-density lipoprotein; LDL: low-density lipoprotein.

### Correlation between elevated TyG indices and SMI incidence

**Fig. 1:**
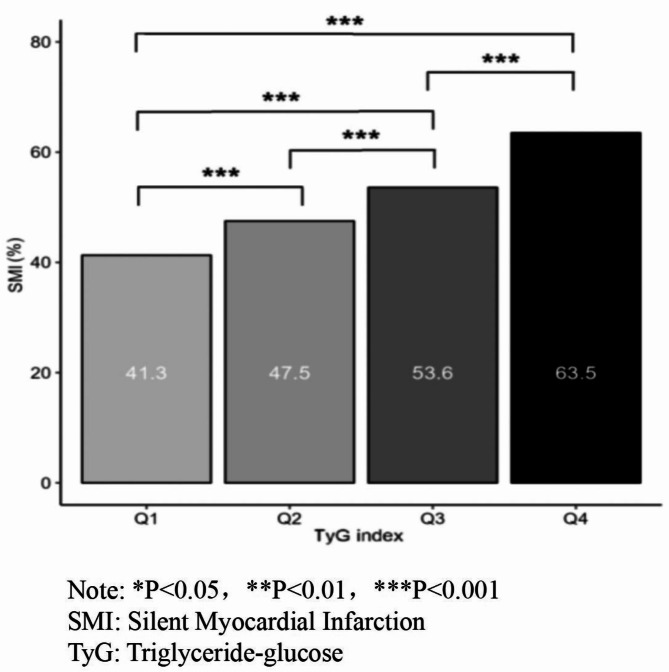
Incidence of SMI in the general population with different TyG levels.

In the community population, individuals with elevated TyG indices showed a higher incidence of SMI. The differences in SMI incidence across the quartile groups of the TyG index were statistically significant (Fig. 1).

### Correlation between the TyG index and SMI risk

**Fig. 2:**
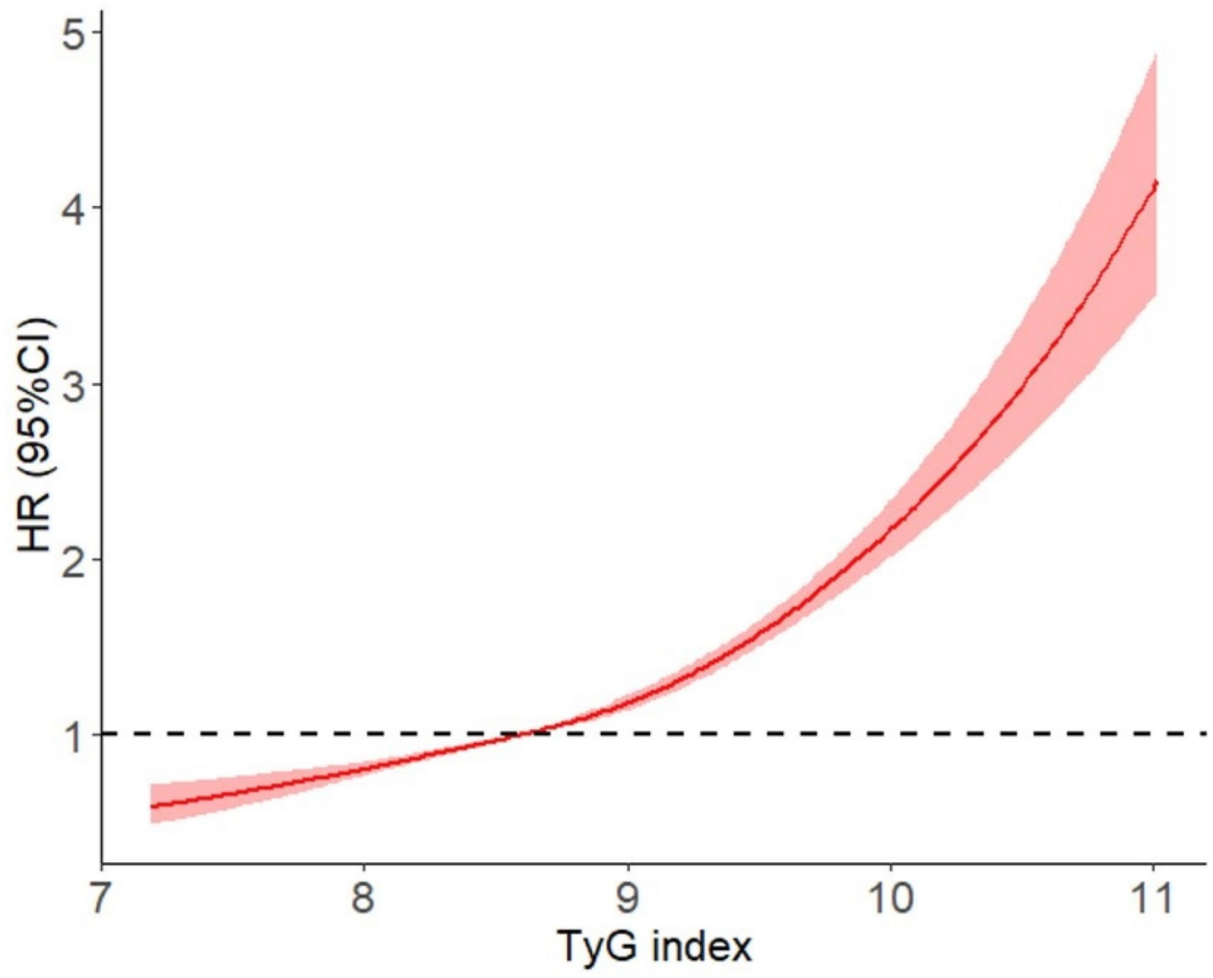
Correlation of the different TyG index levels with the SMI risk.

Restricted cubic spline analysis demonstrated a significant positive association between increased TyG index levels and an elevated risk of SMI. Participants with higher TyG indices had a greater likelihood of developing SMI (Fig. 2).

### Association between the TyG index and survival rates

**Fig. 3:**
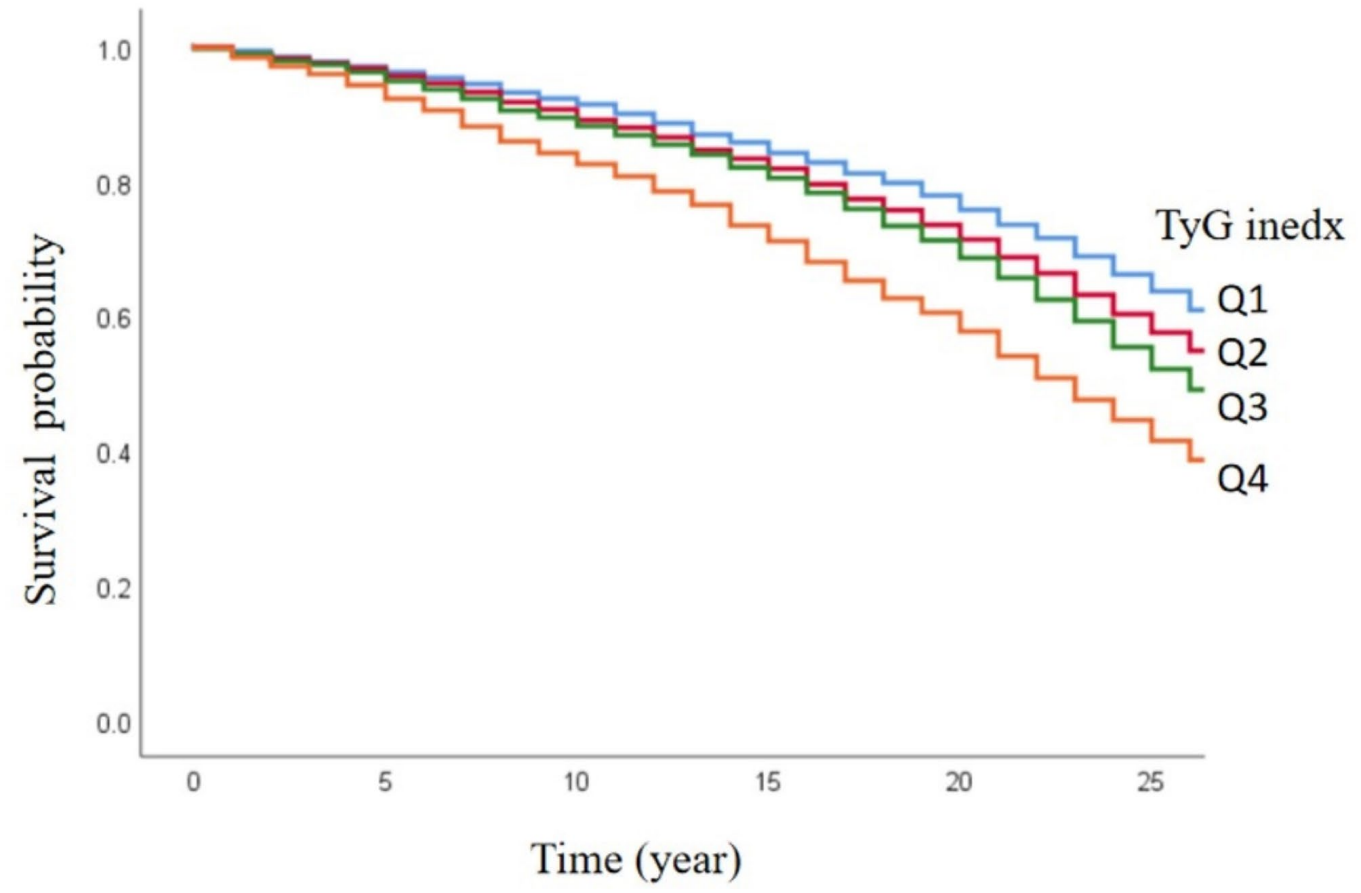
Kaplan–Meier survival analysis of the SMI events among the four groups with different TyG index levels.

Kaplan-Meier survival analysis demonstrated that individuals in the community population with higher TyG indices had the lowest cumulative survival rates for SMI events (Fig. 3).

### Analysis of the TyG index and SMI risk

To evaluate the relationship between the TyG index and the risk of SMI, four distinct models were formulated, as presented in Table 2. In Model 1 (univariate Cox regression), a positive association was found, with higher TyG index quartiles (Q2, Q3, and Q4) showing progressively higher risks of SMI compared to Q1. In Model 2, after adjusting for age, sex, and BMI, though it was weaker for Q2, the positive association remained. In Model 3, after further adjusting for factors such as heart rate, alcohol consumption, smoking status, history of hypertension, and diabetes, the strength of the association was weakened, particularly for Q2 and Q3. In Model 4, after adjusting for some biochemical markers, the strength of the association reduced, with the P-value approaching non-significance. Across all models, a positive correlation was observed between higher TyG index levels and increased SMI risk. However, the strength of this association diminished as additional covariates were introduced.

**Table 2:** Connection between TyG index and SMI risk.

Item	Model 1		Model 2		Model 3		Model 4	
	HR (95%CI)	*P*	HR (95%CI)	*P*	HR (95%CI)	*P*	HR (95%CI)	*P*
Q1 (*n* = 3609)	1	0.000	1	0.000	1	0.000	1	0.000
Q2 (*n* = 3614)	1.21 (1.13–1.30)	< 0.001	1.05 (0.98–1.13)	0.160	1.00 (0.93–1.07)	0.988	0.98 (0.91–1.05)	0.520
Q3 (*n* = 3604)	1.42 (1.33–1.52)	< 0.001	1.16 (1.08–1.24)	<0.001	1.06 (0.99–1.13)	0.115	1.02 (0.95–1.09)	0.651
Q4 (*n* = 3393)	1.94 (1.82–2.08)	<0.001	1.47 (1.37–1.57)	<0.001	1.12 (1.04–1.20)	0.003	1.09 (1.00-1.18)	0.056
TyG	1.61 (1.55–1.68)	< 0.001	1.38 (1.33–1.44)	< 0.001	1.11 (1.06–1.16)	< 0.001	1.09 (1.03–1.15)	0.002

Note: Model 1: unadjusted;

Model 2: adjusted for gender, age, and BMI;

Model 3: adjusted for gender, age, BMI, heart rate, drinking, smoking, hypertension and diabetes;

Model 4: adjusted for gender, age, BMI, heart rate, drinking, smoking, hypertension, diabetes, hyperlipidemiaand WBC count, neutrophil count, TC.

### Subgroup analysis

Subgroup analysis revealed that the TyG index was significantly and independently associated with the risk of SMI in the subgroups of sex, BMI, smoking, drinking, diabetes, hypertension, hyperglycemia, and hyperlipidemia (Table 3).

**Table 3:** Interaction analysis.

Grouping	Subgroup	HR (95%CI)	*P* value	Interaction *P*-value
Sex				< 0.001
	Female	1.802 (1.708, 1.901)	< 0.001	
	Male	1.343 (1.269, 1.422)	< 0.001	
Age				0.0001
	Age < 60 years	1.623 (1.547,1.702)	< 0.001	
	Age ≥ 60 years	1.385 (1.292,1.486)	< 0.001	
BMI				0.8961
	BMI < 30 kg/m^2^	1.573 (1.497,1.653)	< 0.001	
	BMI ≥ 30 kg/m^2^	1.580 (1.477,1.691)	< 0.001	
Drinking				0.0509
	Non-drinker	1.67 (1.581, 1.763)	< 0.001	
	Drinking status	1.544 (1.462, 1.631)	< 0.001	
Smoking				< 0.001
	Non-smoker	1.778 (1.666, 1.897)	< 0.001	
	Smoking status	1.471 (1.401, 1.545)	< 0.001	
Hypertension				0.5218
	No hypertension	1.528 (1.452, 1.608)	< 0.001	
	Hypertension	1.479 (1.389, 1.575)	< 0.001	
Diabetes				0.0283
	No diabetes	1.418 (1.35, 1.488)	< 0.001	
	Diabetes	1.256 (1.15, 1.373)	< 0.001	
Hyperlipidemia				0.0002
	No hyperlipidemia	1.89 (1.735, 2.059)	< 0.001	
	Hyperlipidemia	1.57 (1.487, 1.657)	< 0.001	

### Effects of mediating factors

Mediation analysis showed that sex, BMI, hypertension, and diabetes significantly influenced the risk of SMI (*P* < 0.001). Diabetes had a total effect on the SMI risk of 1.8, with nearly equal direct and mediating effects (52.7% and 47.3%, respectively). Hyperlipidemia had the highest total effect on SMI risk, at 1.95, but its mediating effect was minimal (0.4%) and non-significant (*P* = 0.91). These results are detailed in Table 4.

**Table 4:** Effects of mediating factors.

	Effect	Effect size	Effect size 95%CI	Relative mediation effect	*P*-value
Sex	Total effects	1.79	1.76–1.94	100.00%	<0.001
	Direct effects	1.73	1.69–1.87	93.80%	<0.001
	Mediating effects	1.04	1.03–1.04	6.20%	0.031
BMI	Total effects	1.77	1.68–1.90	100.00%	<0.001
	Direct effects	1.70	1.62–1.81	93.10%	<0.001
	Mediating effects	1.04	1.04–1.05	6.90%	0.022
Hypertension	Total effects	1.78	1.74–1.83	100.00%	<0.001
	Direct effects	1.62	1.59–1.65	83.50%	<0.001
	Mediating effects	1.1	1.09–1.11	16.50%	<0.001
Diabetes	Total effects	1.8	1.77–1.83	100.00%	<0.001
	Direct effects	1.362	1.33–1.37	52.70%	<0.001
	Mediating effects	1.32	1.30–1.37	47.30%	<0.001
Hyperlipidemia	Total effects	1.95	1.93-2.00	100.00%	<0.001
	Direct effects	1.95	1.94–2.02	99.60%	<0.001
	Mediating effects	1.00	0.98–1.01	0.40%	0.91

## Discussion

The present study delved into the potential association between the TyG index and the incidence of SMI within a community-based, prospective cohort design. The results underscore a direct and robust correlation between elevated TyG indices and an augmented risk of SMI, which persists as a significant predictor even subsequent to adjustments for multiple confounding factors. Notably, the TyG index emerges as an independent marker of SMI, with individuals in the highest quartile exhibiting the poorest survival outcomes in terms of SMI events. Subgroup analyses reinforce this robust association, revealing consistency across diverse demographic and clinical profiles, such as sex, age cohorts, smoking habits, diabetic status, and hyperlipidemia. Mediation analysis disentangles the underlying mechanisms, revealing diabetes and hypertension as the primary mediators of the TyG index-SMI risk relationship. Conversely, hyperlipidemia exerts its greatest influence on SMI risk through direct pathways, with minimal mediation through the TyG index. Although participants’ health status (such as smoking and diabetes) was recorded multiple times during the follow-up, other pathological processes (such as new chronic diseases, progression of comorbidities, or new medication use) may not have been fully recorded or updated, and these unmeasured changes could affect the prognosis. Despite adjusting for covariates, unmeasured factors (such as subclinical inflammation, metabolic abnormalities, or psychological factors) could still influence patient outcomes, introducing bias and thereby limiting the generalizability and causal inference of the results.

Prior investigations have established the TyG index as an independent indicator to cardiovascular disease (CVD) risk. For example, a Koreanprospective cohort study underscored the TyG index’s predictive prowess for incident CVD^15^. Among non-diabetic adults, an elevated TyG index emerged as a potent harbinger of ischemic heart disease, underscoring its potential as an early, preclinical marker of cardiovascular risk^23^. A meta-analysis of 20,403 participants across cohort studies validated the TyG index as an independent prognosticator of major adverse cardiovascular and cerebrovascular events, as well as all-cause mortality in MI patients^24^. An additional meta-analysis fortified this notion, revealing that a high TyG index is associated with heightened risk, severity, and poorer outcomes in coronary artery disease^25^. While these studies collectively underscore the TyG index’s link to diverse CVD manifestations^23,26–29^, a dearth of research specifically exploring its relationship with silent myocardial infarction (SMI) remains. This knowledge gap has hindered the TyG index’s broader application in comprehensive CVD risk evaluation. Our study bridges this divide by elucidating a distinct association between elevated TyG index levels and an increased SMI risk, thereby positioning the TyG index as a valuable predictive tool for SMI. These findings suggest that the TyG index could be used for early risk stratification and preventive strategies in the general population, helping to complement and enhance the accuracy of existing risk assessment tools.

The exact mechanisms through which the TyG index impacts the occurrence of SMI remain unclear. It is likely linked to metabolic and vascular disturbances caused by IR, which elevates the risk of cardiovascular events, including SMI^30^. First, a higher TyG index reflects more severe IR. IR leads to various metabolic disturbances that can impair myocardial blood flow, potentially resulting in SMI. Moreover, the TyG index, as a dependable marker of IR, is linked to the release of inflammatory mediators that damage endothelial cells and disrupt endothelial function. This imbalance may contribute to the formation of atherosclerotic plaques, offering a potential mechanism connecting the TyG index to the development of SMI^14,31–33^. Moreover, an elevated TyG index indicates higher levels of blood glucose and triglycerides, suggesting a disturbed metabolic state. This metabolic dysfunction, which is closely related to IR, can lead to dyslipidemia, which is another major risk factor for CVD. Therefore, the TyG index may contribute to SMI risk through multiple interrelated metabolic and inflammatory pathways.

Our research revealed that SMI occurrence was more prevalent among females, a finding consistent with those of previous studies^5^. In the subgroup analysis, the odds ratio (OR) for SMI in females was 1.647, significantly higher than the OR for males at 1.243 (*P* < 0.001). This highlights the important role that sex plays in the development of SMI, with females being at a higher risk than males. The sex disparity in SMI risk may be attributed to physiological, metabolic, and hormonal differences. In particular, reduced estrogen levels in middle-aged and older females may diminish estrogen’s effects on ion channel activities in sensory neurons and rapid pain modulation via estrogen receptors. This reduction could raise pain thresholds, making females less sensitive and more tolerant to pain than males, potentially leading to undetected SMI events^34,35^. This finding suggests that the TyG index could be especially useful for assessing SMI risk in females. While hypertension, diabetes, and hyperlipidemia are considered significant cardiovascular risk factors, their impact on the association between the TyG index and SMI varies considerably. In particular, the risk of SMI appears to be reduced in diabetic patients, possibly due to ongoing diabetes management that helps mitigate the risk of SMI.

Although hyperlipidemia is typically considered a risk factor for CVD, its mediating role in the relationship between the TyG index and SMI appears to be minimal. This suggests that the influence of hyperlipidemia on the risk of SMI primarily occurs through direct pathways rather than being mediated by the TyG index. However, the TyG index may play a more significant role in this context, possibly because it is closely associated with lipid metabolism disorders and effectively captures the main impact of hyperlipidemia on the risk of SMI. Other metabolic abnormalities included in the TyG index may overshadow the mediating effect of hyperlipidemia, thereby reducing its impact. These results highlight the significance of the TyG index as a comprehensive marker of metabolic disturbances and their link to SMI risk. Additional research is necessary to clarify the mechanisms and interactions between the TyG index and SMI, potentially offering new perspectives for SMI prevention and treatment.

This study faced some limitations. First, the TyG index was not consistently monitored during the follow-up period, preventing analysis of how changes in the TyG index might influence SMI risk. Secondly, the specific causes of SMI were not investigated, which limited our ability to explore how the TyG index relates to various SMI etiologies. Additionally, the study’s findings are based on a population from various communities in the United States and have not been confirmed in other geographical areas. Furthermore, the severity of the SMIs identified was not evaluated in this research. Lastly, The TyG index inherently includes measurements of glucose and triglycerides, and incorporating diabetes and hyperlipidemia as adjustment variables in the model may lead to collinearity issues, thereby affecting the independent interpretation of the association between the TyG index and SMI risk. This could potentially weaken the model’s ability to evaluate the standalone effect of the TyG index. Nonetheless, we chose to include these variables to achieve a more comprehensive control of confounding factors.

## Conclusions

This prospective cohort study in a community population identified an independent association between elevated TyG indices and a higher risk of SMI. This association enables healthcare providers to formulate more specific preventive measures based on the TyG index, introducing novel strategies for early detection and intervention in cardiovascular health, with the ultimate goal of reducing the occurrence of cardiovascular events in patients.

## Data Availability

The data that support the findings of this study are available on request from the corresponding author.
